# High thermoelectric performance in metallic NiAu alloys via interband scattering

**DOI:** 10.1126/sciadv.adj1611

**Published:** 2023-09-15

**Authors:** Fabian Garmroudi, Michael Parzer, Alexander Riss, Cédric Bourgès, Sergii Khmelevskyi, Takao Mori, Ernst Bauer, Andrej Pustogow

**Affiliations:** ^1^ Institute of Solid State Physics, TU Wien, 1040 Vienna, Austria.; ^2^ International Center for Young Scientists (ICYS), National Institute for Materials Science, Tsukuba, Japan.; ^3^ Research Center for Computational Materials Science and Engineering, TU Wien, 1040 Vienna, Austria.; ^4^ International Center for Materials Nanoarchitectonics (WPI-MANA), National Institute for Materials Science, Tsukuba, Japan.; ^5^ Graduate School of Pure and Applied Sciences, University of Tsukuba, Tsukuba, Japan.

## Abstract

Thermoelectric materials seamlessly convert thermal into electrical energy, making them promising for power generation and cooling applications. Although historically the thermoelectric effect was first discovered in metals, state-of-the-art research focuses on semiconductors. Here, we discover unprecedented thermoelectric performance in metals and realize ultrahigh power factors up to 34 mW m^−1^ K^−2^ in binary Ni*_x_*Au_1–*x*_ alloys, more than twice larger than in any bulk material above room temperature, reaching *zT*_max_ ∼ 0.5. In metallic Ni*_x_*Au_1–*x*_ alloys, large Seebeck coefficients originate from electron-hole selective scattering of Au *s* electrons into more localized Ni *d* states. This intrinsic energy filtering effect owing to the unique band structure yields a strongly energy-dependent carrier mobility. While the metastable nature of the Ni-Au system as well as the high cost of Au pose some constraints for practical applications, our work challenges the common belief that good metals are bad thermoelectrics and presents an auspicious route toward high thermoelectric performance exploiting interband scattering.

## Teaser

Metallic NiAu alloys display intriguing thermoelectric properties enabled by strong intrinsic interband scattering.

## INTRODUCTION

**Fig. 1.**
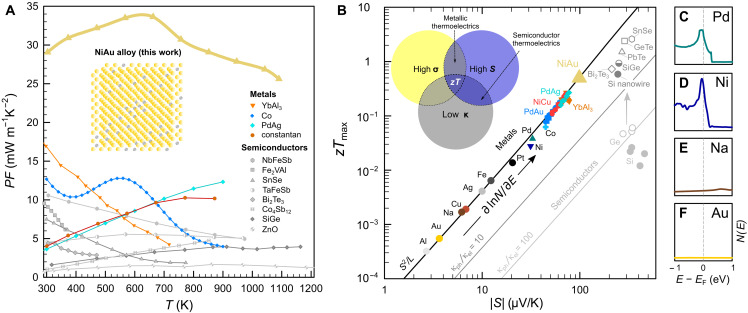
Thermoelectric performance of NiAu alloys compared to today’s best thermoelectrics. (**A**) Power factor of Ni_0.1_Au_0.9_ and various semiconducting (*45*–*52*) and metallic (*17*, *29*, *32*, *34*) systems. (**B**) Wiedemann-Franz plot of thermoelectrics: universal scaling *zT* ∝ *S*^2^/*L* defines the TE performance in metals. Conventional semiconductors like Si and Ge exhibit *zT* orders of magnitude below *S*^2^/*L* as the lattice thermal conductivity is much larger than the electronic contribution, κ_ph_ ≫ κ_el_. Despite extremely low κ_ph_, state-of-the-art semiconductors still have several times lower *zT* compared to the metallic limit *S*^2^/*L*, where TE performance is optimal for a given value of *S*. DOS *N*(*E*) for metallic elements with (**E** and **F**) delocalized *s* and (**C** and **D**) more localized *d* states near *E*_F_. Because *s-d* scattering is proportional to *N*(*E*), ∂ln*N*(*E*)/∂*E* determines *zT* ∝ *S*^2^ in metals. A detailed list of data and references (*4*, *5*, *29*, *32*, *34*, *44*, *53*–*66*) are shown in table S1.

Thermoelectric (TE) materials have garnered considerable interest for various applications, such as thermometry, refrigeration, and power generation (*1*). After Ioffe’s proposal in the 1930s (*2*), most investigations focused on semiconductors, with the general idea being that a bandgap is required to obtain strong asymmetry in the electronic density of states (DOS) *N*(*E*) around the Fermi energy *E*_F_, a prerequisite for a large Seebeck effect. This predominant notion has confined research to a narrow fraction of known materials, neglecting a myriad of metallic systems. Despite remarkable progress achieved by adhering to this paradigm, the most substantial improvement of the figure of merit *zT* = (*S*^2^σ/κ)*T* has been obtained through the reduction of the thermal conductivity κ via phonon engineering (*3*–*6*), which is inherently limited. Therefore, identifying innovative enhancement principles for the power factor, *PF* = *S*^2^σ, is of great interest, especially for power generation (*7*, *8*) or the emerging field of active cooling (*9*–*11*). Here, *S* and σ are the Seebeck coefficient and electrical conductivity, respectively. These two electronic transport properties are entangled such that an increase of σ also decreases *S* and vice versa. While numerous strategies to tackle this problem and enhance the power factor have been proposed (*12*–*15*), the simultaneous realization of high *S* and σ remains one of the toughest challenges for TE research, restraining widespread use of TE technology. Here, we report ultrahigh power factors >30 mW m^−1^ K^−2^ in binary Ni*_x_*Au_1–*x*_ metallic alloys, vastly exceeding those of any known bulk material above room temperature (Fig. 1A) as well as *zT* values up to ~0.5, higher than in any other metallic system (Fig. 1B). Our electronic structure calculations reveal that this is the result of a strongly energy-dependent scattering rate τ^−1^ arising from a steep gradient of Ni *d* states around *E*_F_. While *s*-type holes are scattered into more localized Ni *d* states below *E*_F_, the mobility remains high for conduction electrons above *E*_F_. Furthermore, we emphasize that in good metallic conductors, where thermal transport is largely dictated by the electronic contribution, only the Seebeck coefficient has to be tuned to enhance the figure of merit, which simplifies to *zT* = *S*^2^/*L* due to the Wiedemann-Franz law (Fig. 1B), where *L* represents the Lorenz number. Hence, our work constitutes a promising paradigm in TE research, circumventing the multidimensional optimization problem in semiconductors that involves balancing the trade-offs between *S*, σ, and κ.

## RESULTS

**Fig. 2.**
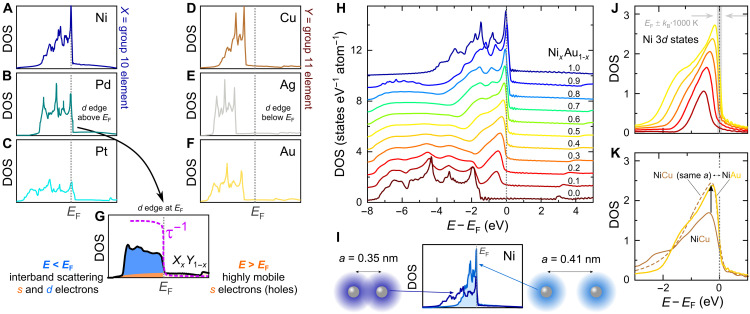
Electron-hole selective *s–d* scattering in binary metallic alloys. (**A** to **F**) Electronic DOS *N*(*E*) of group 10 (*X*) and 11 (*Y*) elements. While the filled *d* states lie more than 1 eV below *E*_F_ for *Y*, the partial *d* occupation in *X* yields large DOS at *E*_F_; paramagnetic *N*(*E*) shown for *X *= Ni. (**G**) The steep slope of *N*(*E*_F_) involves strong *s-d* scattering at *E* < *E*_F_, resulting in a steep gradient of the scattering rate τ^−1^ across *E*_F_. (**H**) In binary alloys, such as Ni*_x_*Au_1–*x*_, the *d* edge of *X* atoms is successively tuned through *E*_F_ upon changing *x*. (**I**) Increasing the lattice spacing of Ni to *a*_Au_ =0.41 nm reduces the bandwidth and steepens the *d* edge compared to elemental Ni (*a*_Ni_ = 0.35 nm). (**J**) Partial DOS of Ni *d* states in Ni*_x_*Au_1–*x*_; gray bar indicates a thermal energy of 1000 K around *E*_F_. (**K**) Ab initio calculations yield similar *N*_Ni−*d*_(*E*) for NiCu and NiAu when the same lattice parameter is used, identifying chemical pressure as the pivotal parameter.

**Fig. 3.**
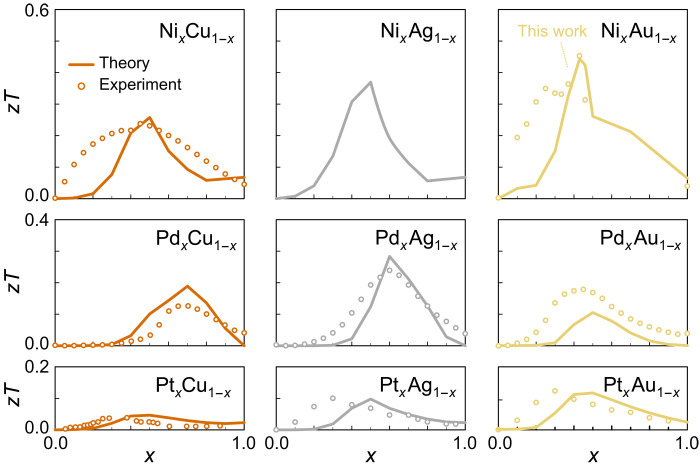
*zT* from *s-d* scattering compared to experiment. The composition-dependent *zT* at 1000 K, calculated within the theoretical framework of *s-d* scattering τ^−1^ ∝ *N*(*E*), agrees well with experimental results on *X_x_Y*_1−*x*_ (*X *= Ni, Pd, Pt; *Y *= Cu, Ag, Au) from literature (*29*, *44*, *54*) and this work despite the lack of free model parameters. The highest *zT* is reached for Ni alloys due to the more localized 3*d* states compared to the 4*d* and 5*d* bands of Pd and Pt. Among group 11 elements, Au alloys show the highest performance due to the negative chemical pressure further reducing the bandwidth of Ni *d* states.

**Fig. 4.**
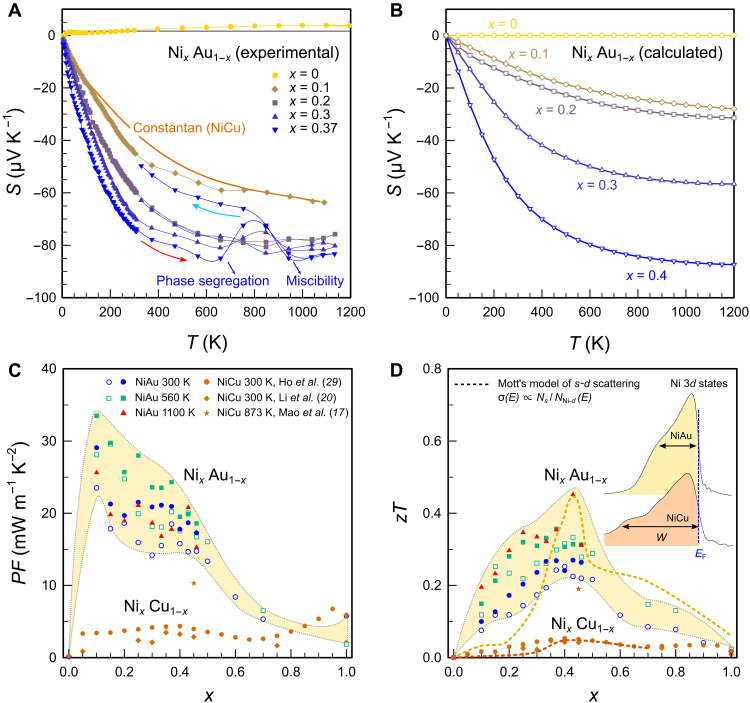
Thermoelectric properties of the Ni-Au system. (**A**) Temperature-dependent Seebeck coefficient of selected Au-rich Ni*_x_*Au_1–*x*_ alloys. The Seebeck coefficient of constantan (Ni_0.45_Cu_0.55_) is shown for reference. Au-rich samples with *x* < 0.3 are (meta)stable and show no hysteresis even up to 1100 K. With increasing Ni concentration, however, phase segregation occurs during the measurement (indicated by arrows), followed by reentrant miscibility above the critical temperature of the solid solution. (**B**) Temperature-dependent Seebeck coefficient calculated from electronic DOS within the framework of *s-d* scattering. (**C**) Composition-dependent power factor of the Ni-Au system at various temperatures within the single-phase regime. Data have been obtained at TU Wien, Austria (unfilled symbols) and the National Institute for Materials Science (NIMS), Japan (filled symbols). The power factor of the well-known Ni-Cu system is plotted for comparison (*17*, *20*, *29*). (**D**) Composition-dependent figure of merit of Ni-Au and Ni-Cu binary alloy systems. Dashed lines are theoretical calculations, where the interband scattering rate is estimated from the DOS within the paramagnetic regime (see Materials and Methods). No free parameters were used in the model. Inset shows that the bandwidth of Ni 3*d* states decreases for NiAu with respect to NiCu. The steeper slope of the Ni *d* edge leads to a strongly increased TE performance of NiAu alloys.

In the following, we will demonstrate that high TE performance above room temperature can also be realized in materials without a gap in the DOS around *E*_F_, i.e., in metals. Figure 1 (C to F) shows the DOS near *E*_F_ for several metallic elements. Alkali metals like Na as well as transition metals such as Cu, Ag, and Au have a symmetric distribution *N*(*E*) around *E*_F_ due to their partly filled *s* orbitals with a large bandwidth *W* of several electron volts. In these systems, *zT* is orders of magnitude smaller compared to transition metals with sharp *d* states near *E*_F_, like Ni or Pd (Fig. 1B). We will show that the increased TE performance originates from the energy dependence of τ^−1^ upon *s* → *d* scattering and quantitatively estimate *zT* directly from *N*(*E*). To examine this relationship for the different elements, we constructed a periodic table of densities of states (fig. S1), which summarizes the electronic structures of solid elements and can be used as a starting point for future studies searching for high TE performance in metals. The concept of *s-d* scattering dates back to Mott’s work in 1935 describing the electronic structure of the Pd-Ag system (*16*). In PdAg alloys, high-velocity Ag *s*-type carriers scatter into rather immobile Pd *d* states, which increases the residual resistivity and also the diffusion term of the thermopower. Similarly, an unusually high Seebeck effect is found in isovalent NiCu alloys, exploited in thermocouples. Over the last few years, there have been several attempts to enhance the TE properties of NiCu alloys (*17*–*21*), yet a clear strategy to effectively optimize the electronic structure and thereby boost the TE performance is still lacking. Thus, with some exceptions (*22*–*24*), metals have been largely overlooked by the majority of the TE community, dismissing interband scattering as a potential route to design thermoelectrics. In our quest for high-performance metallic thermoelectrics, we investigated all combinations of binary alloys between group 10 and 11 transition metals and estimated the TE performance of these systems based on the evolution of their electronic structure. Figure 2 (A to F) shows *N*(*E*) of *X *= Ni, Pd, Pt and *Y *= Cu, Ag, Au in their pure form. The *d* levels are not fully occupied for *X* and, in the case of Ni, *N*(*E*_F_) surpasses the Stoner criterion *JN*(*E*_F_) > 1 (*J* is the exchange interaction), giving rise to ferromagnetism below 633 K. For *Y*, the *d* states are completely filled and lie more than 1 eV below *E*_F_. The small featureless DOS of high-velocity *s* and *p* electrons around *E*_F_ makes Cu, Ag, and Au the best metallic conductors. In the solid solution *X_x_Y*_1−*x*_, the *d* edge of the *X* atoms is shifted across *E*_F_ with decreasing *x*. Our ab initio calculations on Ni*_x_*Au_1−*x*_ demonstrate the successive pileup of Ni *d* states below *E*_F_ (Fig. 2, H and J), which is similar for all other combinations *X_x_Y*_1−*x*_ as shown in fig. S2. As already postulated by Mott (*16*), the more localized and much less conductive Ni *d* states (σ*_d_* ≪ σ*_s_*) hardly contribute to the Seebeck coefficient, which is a weighted average of the *s*- and *d*-band contributions *S* = (*S_s_* σ*_s_* + *S_d_* σ*_d_*)/(σ*_s_* + σ*_d_*) ≈ *S_s_*. Instead, they induce strong interband scattering for the *s* electrons in the overlapping energy range, which heavily influences the Seebeck coefficient. In first approximation, the Seebeck coefficient is given by the well-known Mott formula

$$S_{s}=-\frac{\pi^{2}k_{B}^{2}}{3e}T{\left(\frac{\partial \ln N_{s}}{\partial E}-\frac{\partial \ln\tau_{s-d}^{-1}}{\partial E}\right)}_{E=E_{F}}$$ (1)

where *N_s_* is the DOS for the conducting *s* electrons and $\tau_{s-d}^{-1}$ is the interband scattering rate for mobile *s* electrons scattering into Ni *d* states. The large bandwidth of *s* states yields negligibly small ∂ln*N_s_*/∂ *E* in *X_x_Y*_1−*x*_ metallic alloys. The second term in Eq. 1 arising from the energy-dependent scattering rate is commonly neglected in semiconductors but dominates the TE transport in such metallic alloys. This can be easily rationalized by Fermi’s golden rule according to which the phase space of *s-d* scattering scales with *N*_Ni−*d*_, leading to a pronounced energy dependence of the scattering rate near the Fermi level $\tau_{s-d}^{-1}\propto N_{\mathrm{Ni}-d}$ (see the dashed magenta line in Fig. 2G). Consequently, hole-type carriers contribute much less to the overall charge transport than conduction electrons above *E*_F_, resulting in sizeable values of the Seebeck coefficient. To identify the most promising *X_x_Y*_1−*x*_ alloys, we calculated the temperature-dependent Seebeck coefficient of *X_x_Y*_1−*x*_ by solving the respective transport integrals (see Materials and Methods) using a simple *s-d* scattering model where the energy-dependent transport function is estimated as σ(*E*) ∝ 1/*N*(*E*) from Fermi’s golden rule. A similar type of analysis was previously performed just for the Pd-Ag system (*25*); yet, more accurate first-principles calculations have to be pursued in future works using the Kubo-Greenwood formalism (*26*–*28*). Nonetheless, Fig. 3 shows that *zT* ≈ *S*^2^/*L* of *X_x_Y*_1−*x*_ yields remarkable agreement with experimental results obtained here and in literature (*29*) despite the simplicity and lack of any free parameters in our model. We have accounted for the deteriorating effect of electron-phonon scattering on the Seebeck coefficient, becoming prominent at *T* > 300 K, via the Nordheim-Gorther rule (Materials and Methods) (*30*, *31*). From Fig. 3, it becomes clear that Ni is the most promising group 10 element for the design of TE metals, owing to more localized and sharper *d* states (Fig. 2, A to C) and also lower cost than Pd and Pt. Moreover, among Ni alloys with group 11 elements, Ag and Au exhibit the highest *zT*, which we attribute to the larger lattice parameter compared to Cu (no data exist for Ni-Ag due to their immiscibility). As a consequence of this negative chemical pressure, there is less overlap between Ni *d* orbitals, leading to even more localized states and, hence, larger *S* ∝ ∂ln*N*_Ni−*d*_/∂*E* (Fig. 2, I and J). After this comprehensive assessment, we experimentally studied the TE properties of the binary Ni-Au system over the whole temperature (4 to 1100 K) and composition range. In our endeavor, we had to overcome the metastable nature of Ni*_x_*Au_1–*x*_. Its peculiar phase diagram involves a miscibility gap at low temperatures due to a large size mismatch of the lattice constants and a narrow region of solid solubility at high temperatures (fig. S4). By quenching NiAu samples in water, we succeeded in obtaining the desired fcc phase at ambient temperatures. As seen in Fig. 4A, a heating-cooling cycle of a sample with 37 atomic % (at %) Ni reestablishes the mixed phase with Ni-rich and Au-rich regions, evident from a reduction of ∣*S*∣. To evade the phase-segregated regime, we studied the single-phase alloys at *T* ≥ 1000 K, where we achieve the highest *zT* ∼ 0.5 (Fig. 4D), corresponding to *S* ∼ 94 μV/K. While the peak values for *PF* and *zT* are realized at 10 and 43 at % Ni, respectively, the TE performance surpasses that of constantan (NiCu) by several times over the entire composition range—a manifestation of the different electronic bandwidth of Ni atoms in Cu versus Au lattices. A closer assessment of *S*(*T*) in Fig. 4A yields a linearly increasing Seebeck coefficient that saturates at high temperatures, in good agreement with our theoretical estimation in Fig. 4B. Note the quantitative agreement with experimental data at *x* ∼ 0.4, where the electron-hole asymmetry of *s-d* scattering is most pronounced; deviations between experiment and theory become larger further away from the optimum. We also note that alloys with *x* < 0.3 hardly show hysteretic behavior of *S*(*T*) upon repeated thermal cycles, indicating that these become quasistable compositions with probably very large timescales of phase dissociation (fig. S7). The SD from the mean value for five consecutive measurement curves was less than 3% for the Seebeck coefficient and <5% for the power factor at all measured temperatures (fig. S7). Measurements were independently carried out on numerous samples in different laboratories [TU Wien, National Institute for Materials Science (NIMS)], confirming the validity and reproducibility of our data.

## DISCUSSION

The main finding of our study is the record-high power factors in Ni*_x_*Au_1–*x*_, reaching peak values of 34 mW m^−1^ K^−2^ at 560 K and 29 mW m^−1^ K^−2^ at room temperature. For *x* = 0.1, we find an average *PF *∼ 30 mW m^−1^ K^−2^ over an extremely broad temperature range 300 to 1100 K. Such colossal values above room temperature exceed those of state-of-the-art semiconductors by an order of magnitude (Fig. 1A) and are even much larger than the giant power factors in strongly correlated *f* electron systems like YbAl_3_ (*32*) and CePd_3_ (*33*) or the magnon-drag metal cobalt (*34*). This implies a great potential for electrical power generation, especially if the heat source is large (*7*), although the high cost of Au poses constraints on widespread use in industry. Another potential application for materials with high *PF* is the field of active cooling, where the Peltier effect is used to pump heat from the hot side toward ambient temperature. It was shown that, in such cases, the relevant quantity to be maximized is the so-called effective thermal conductivity $\kappa_{\mathrm{eff}}=\kappa +PF\;T_{H}^{2}/(2\text{Δ}T)$, with the second term being the active cooling contribution (*9*). Recent works showed that elemental Co (*10*) (κ_eff_ = 780 W m^−1^ K^−1^) and carbon nanotube fibers (*11*) (1190 W m^−1^ K^−1^) can exceed the thermal conductivity of pure copper for Δ*T *= 1 K (*10*). Under the same conditions, we obtain κ_eff_ ∼ 1400 W m^−1^ K^−1^ for Ni_0.1_Au_0.9_, substantially exceeding κ_eff_ of any material reported so far. Because of their great mechanical properties (high ductility and flexibility) and because they are very easy to manufacture and produce, these alloys are ideal candidates for active cooling applications, e.g., in integrated circuits. Ductile metallic alloys are also interesting for flexible devices such as power supplies for wearable electronics, which convert heat from the human body into electricity. So far, ductile thermoelectrics for such applications have only been achieved in organic semiconductors (*35*) with *zT* ∼ 0.42 or recently discovered AgCu(Se,S,Te)-based ductile semiconductors (*36*) with *zT* ∼ 0.45 at *T* = 300 K. In comparison, simple binary NiAu alloys already show *zT* ∼ 0.3 near room temperature and extremely high ductility, demonstrating that metallic alloys are suitable candidates for future study on flexible thermoelectrics. Apart from niche applications such as thermocouples, metals have been discarded as TE materials, due to their usually small Seebeck effect. However, by combining the concepts of interband scattering and bandwidth tuning, a tool commonly utilized in correlated electron research, we have constructed a roadmap that can accurately predict high TE performance in metallic alloys. Moreover, we have experimentally confirmed this approach in binary NiAu alloys as a proof of concept and discovered unprecedentedly high power factors as well as the largest *zT* ever reported for a metal, both over a broad temperature range. We emphasize that the enhanced TE performance via interband scattering is not bound to the specific elements Ni or Au, but is rather a general concept. Theoretical investigations reveal that the performance of metallic alloys can be strongly enhanced by bandwidth tuning via chemical pressure as demonstrated here. Our theoretical predictions indicate that even in pure Ni, an increase of the lattice parameter by ∼16% would result in a marked enhancement of the Seebeck effect, potentially reaching *zT* > 1 at room temperature (fig. S3). From an experimental point of view, further insights into the effects of interband scattering on the TE performance can be obtained by measuring the Nernst effect, which is a direct probe of dynamical quasiparticle lifetimes. Recently, it was demonstrated that the contribution of energy-dependent carrier scattering rates to the Seebeck effect can be estimated from temperature-dependent Nernst and Hall signals (*37*). Moreover, guided by our periodic table of densities of states, we identify other binary systems with similar or even more promising electronic structures such as Ni-Na, Ir-Na, Rh-Na, Rh-K, Ir-K, Rh-Rb, Ir-Rb, Co-Si, Co-Ge, Co-Sn, Co-In, Ni-In, Ni-Sn, Sc-Cu, Sc-Ag, and Sc-Au. In particular, systems comprising transition and alkali metals show ultralocalized states next to *E*_F_ due to the much larger atomic radii of the alkalis leading to very narrow *d* bands upon lattice expansion. In conjunction with the broad *s*-type conduction bands, this implies a great potential for strong interband scattering. Although the *zT* values of state-of-the-art inorganic semiconductors are still several times greater than those reported here, our work presents a major breakthrough in the search for high-performance metallic thermoelectrics at *T* ≥ 300 K and sets the stage for avenues of TE research utilizing interband scattering. Metallic alloys with high TE performance have many advantages compared to their semiconducting counterparts, which often face problems such as high contact resistance at the interface between the TE semiconductor and metal electrodes due to the formation of a Schottky barrier or thermal and mechanical degradation, impeding widespread application of TE devices (*38*, *39*). Because of their high ductility, mechanical strength and superior processability, metallic alloys have a huge potential to expand the field toward TE applications that cannot be realized by conventional inorganic semiconductors, especially if high-performance systems consisting of cheap and abundant elements are realized.

## MATERIALS AND METHODS

### Electronic structure calculation

Ab initio electronic structure calculations of binary metallic alloys were performed within the framework of density functional theory and self-consistent bulk Green-function formalism on the basis of the coherent potential approximation (*40*, *41*) within a dense grid of 75 × 75 × 75 *k*-points (9880 *k*-points in the irreducible Brillouin zone). To treat exchange correlation, the Perdew-Wang parametrization of the local density approximation was used (*42*). The DOS of pure transition metal elements were partly taken from the Materials Project database and partly calculated using the Vienna Ab Initio Simulation Package with similar computational parameters.

### Transport modeling

The total Seebeck coefficient of a multi-band electronic conductor results by weighting the respective contributions of the Seebeck coefficient with the electrical conductivities. Thus, in the case of Ni*_x_*Au_1–*x*_ alloys with primarily two types of charge carriers, *s* electrons with high mobility and the less mobile *d* electrons, the net diffusion thermopower can be written as

$$S=\frac{S_{s}\;\sigma_{s}+S_{d}\;\sigma_{d}}{\sigma_{s}+\sigma_{d}}$$ (2)

The energy-dependent conductivity is given by σ(*E*) ∝ *N*(*E*) τ(*E*), with *N*(*E*) and τ(*E*) being the electronic DOS and relaxation time, respectively. For *s*-like conduction electrons scattering into empty *d* states (*s*-*d* scattering) the electrical conductivity yields σ*_s_* ∝ *N_s_*/*N*_Ni−*d*_ because the scattering rate τ^−1^ ∝ *N*_Ni−*d*_, as given by Fermi’s golden rule. Using the Mott expression, *S* ∝ − *∂*lnσ*/∂E*, as well as σ*_s_* ≫ σ*_d_*, Eq. 2 from above can be rewritten to a first approximation (at low temperatures) as

$$S\propto \frac{-\frac{\partial \ln\sigma_{s}}{\partial E}\sigma_{s}-\frac{\partial \ln\sigma_{d}}{\partial E}\sigma_{d}}{\sigma_{s}+\sigma_{d}}\approx S_{s}\approx -\frac{\partial \ln\sigma_{s}}{\partial E}$$ (3)

Consequently, the Seebeck coefficient is given by the positive logarithmic derivative of the DOS of the Ni *d*-like electrons

$$S_{s}=\frac{\pi^{2}k_{B}^{2}}{3e}T{\left(\frac{\partial \ln N_{\mathrm{Ni}-d}}{\partial E}\right)}_{E=E_{F}}$$ (4)

With *zT* = *S*^2^σ*T*/(*L*σ*T* + κ_ph_) ≈ *S*^2^/*L*, this yields a simple formula for the figure of merit solely depending on natural constants and the DOS. To conduct a more in-depth examination of the TE properties at high temperatures, where the Fermi-Dirac distribution is broadened, the transport integrals must be solved

$$S(T)=\frac{k_{B}}{e}\frac{\int_{-\infty }^{\infty }\sigma (E)\frac{E-\mu }{T}\left(-\frac{\partial f_{0}}{\partial E}\right)dE}{\int_{-\infty }^{\infty }\sigma (E)\left(-\frac{\partial f_{0}}{\partial E}\right)dE}$$ (5)

Here, *f*_0_(*E*, μ, *T*) represents the Fermi-Dirac distribution function, μ is the chemical potential, and σ(*E*) ∝ 1/*N*(*E*) is the transport function. To theoretically estimate the composition-dependent figure of merit of binary metallic alloys comprising transition metals from group 10 and group 11 elements, the Seebeck coefficient of *s-d* scattering was calculated via Eq. 5. The *s*-*d* scattering in *X_x_Y*_1−*x*_ alloys is mainly caused by the elastic impurity scattering at the *X* atoms. However, it is important to consider the various scattering processes when calculating the Seebeck coefficient, particularly at high temperatures where contributions like electron-phonon scattering become more important. To account for these contributions at high temperatures, the Nordheim-Gorther rule (*30*, *31*) was used

$$S(T)=\frac{S_{s-d}\rho_{s-d}+S_{\mathrm{ph}}\rho_{\mathrm{ph}}}{\rho_{s-d}+\rho_{\mathrm{ph}}}\approx \frac{S_{s-d}\rho_{s-d}}{\rho_{s-d}+\rho_{\mathrm{ph}}}$$ (6)

Here, *S*_*s*−*d*_ represents the Seebeck coefficient due to *s-d* scattering, which was calculated by solving the transport integrals shown in Eq. 4 using σ(*E*) ∝ 1/*N*_Ni−*d*_(*E*). ρ_*s*−*d*_ is the resistivity due to *s-d* scattering and *S*_ph_, ρ_ph_ are the contributions due to electron–phonon scattering. In our model, we assumed *S*_*s*−*d*_ ≫ *S*_ph_, ρ_*s*−*d*_ = ρ_0_, and hence, ρ_ph_ = ρ − ρ_0_, due to Mathiessen’s rule, with ρ_0_ being the residual resistivity at low temperatures arising from elastic impurity scattering. ρ_0_ and ρ are obtained from experiments (for Ni-Ag, where no experimental data exist, the resistivity values of the Ni-Cu system were taken). Within this framework, *zT* = *S*^2^/*L* was calculated without any free parameters, yielding very good qualitative and quantitative agreement with experimental high-temperature data. The Lorenz number *L* was estimated from a simple formula *L* = 1.5 + exp(−∣*S*∣/116), which is widely used for TE materials (*43*).

### Sample preparation and characterization

Binary NiAu alloys were prepared by stoichiometrically weighing 99.95% pure Ni and 99.99% pure Au bulk pieces. The small metal pieces were mounted in a copper hearth under argon atmosphere and melted by high-frequency induction heating. The as-cast ingots were cut using a high-speed diamond cutting wheel. Because the Ni-Au phase diagram has a large miscibility gap and a narrow region of solid solubility at high temperatures, the properties of NiAu alloys depend strongly on the synthesis procedure. Samples that are slowly cooled from the melt dissociate into two separate phases, one gold-rich phase and one of almost pure Ni, resulting in two-phase microstructures (see fig. S4). Therefore, structural characterization (x-ray diffraction, scanning electron microscopy, and electron dispersive x-ray analysis) were used to study the microstructure and phase homogeneity of our samples. The crystal structure and phases of all samples were analyzed via x-ray diffraction in a Bragg-Brentano geometry using conventional Cu-Kα radiation. Slowly cooled samples displayed two fcc phases, whereas quenched samples showed a single fcc phase. Quenched samples were measured from different sides (bottom side and top side) to ensure no spatial inhomogeneities were present within the samples. According to the Ni-Au phase diagram, there exists a phase boundary toward a single-phase solid solution at high temperatures at all compositions. To verify that our NiAu samples become single phase at high temperatures within the timescale of the high-temperature TE property measurement, temperature-dependent in situ x-ray diffraction experiments have been performed from room temperature up to 1273 K, sufficiently above the phase transition temperature (see fig. S4). We confirmed that within <30 min, even the slowly cooled two-phase sample with the largest amount of secondary phase transforms into a single fcc phase. The microstructure and composition were investigated by using a scanning electron microscope and energy-dispersive x-ray analysis at the University Service Center for Transmission Electron Microscopy, TU Wien (USTEM), TU Wien. Slowly cooled samples displayed a two-phase microstructure as can be seen in fig. S4. However, macroscopically, the composition as well as the microstructure were confirmed to be consistent and homogeneous across the sample. Hence, this allowed us to use these samples for rapid thermal quenching in water, which resulted in single-phase NiAu alloys of the desired nominal composition.

### Property measurements

TE transport measurements have been performed at different setups at TU Wien in Austria as well as at the National Institute for Materials Science in Japan. Low-temperature investigations (4 to 300 K) of the electrical resistivity and Seebeck coefficient have been performed at our in-house setups at the Institute of Solid State Physics, TU Wien. The resistivity was measured using a four-probe method with an ac resistance bridge (Lakeshore) and an excitation current of 31.6 mA. Thin gold wires were spot welded onto the sample surface in the appropriate geometry and the sample was mounted in a helium bath cryostat. The low-temperature Seebeck coefficient was measured by making use of a toggled heating technique with two constantan-chromel thermocouples that were thermally and electrically contacted to the sample. High-temperature measurements of the Seebeck coefficient and electrical resistivity have been performed by making use of a commercially available setup (ZEM3 by ULVAC) at TU Wien as well as at NIMS. Additionally, the thermal conductivity was measured for a representative sample close to the optimal composition (33 at % Ni) at TU Wien using a LightFlash (LFA 500) diffusivity measurement setup by Linseis. This allowed us to confirm that the thermal conductivity of NiAu alloys is entirely dominated by electronic heat transport κ ≈ κ_el_ (see fig. S8). Owing to the very soft lattice and low Debye temperature of Au (Θ_D_ ≈ 165 K), the lattice contribution of the thermal conductivity makes up for less than 1% of κ in pure Au. In concentrated Au-rich CuAu alloys, which are structurally identical to the NiAu alloys studied here, κ_ph_ is only around 1 W m^−1^ K^−1^ at room temperature and 0.5 W m^−1^ K^−1^ at 1100 K (*44*) (<0.5% of the total thermal conductivity). Thus, the thermal conductivity of NiAu alloys obeys and is accurately described by the well-known Wiedemann-Franz law. Calculating κ_el_ from the Wiedemann-Franz law by using an estimate for the Lorenz number as derived in (*43*) reveals almost perfect agreement with the experimental data (see fig. S8).
