# Supplementary material for: High thermoelectric performance in metallic NiAu alloys via interband scattering
Source: Sci Adv. 2023 Sep 15;9(37):eadj1611. doi: 10.1126/sciadv.adj1611 (PMC10881022; doi:10.1126/sciadv.adj1611)
Supplement: Supplementary file 1 — Figs. S1 to S8 Table S1 [file sciadv.adj1611_sm.pdf]

Science Advances
AAAS

Supplementary Materials for

High thermoelectric performance in metallic NiAu alloys via interband scattering

Fabian Garmroudi *et al.*

Corresponding author: Fabian Garmroudi, fabian.garmroudi@tuwien.ac.at;
Michael Parzer, michael.parzer@tuwien.ac.at; Andrej Pustogow, pustogow@ifp.tuwien.ac.at

Sci. Adv. **9**, eadj1611 (2023)
DOI: 10.1126/sciadv.adj1611

This PDF file includes:

Figs. S1 to S8
Table S1

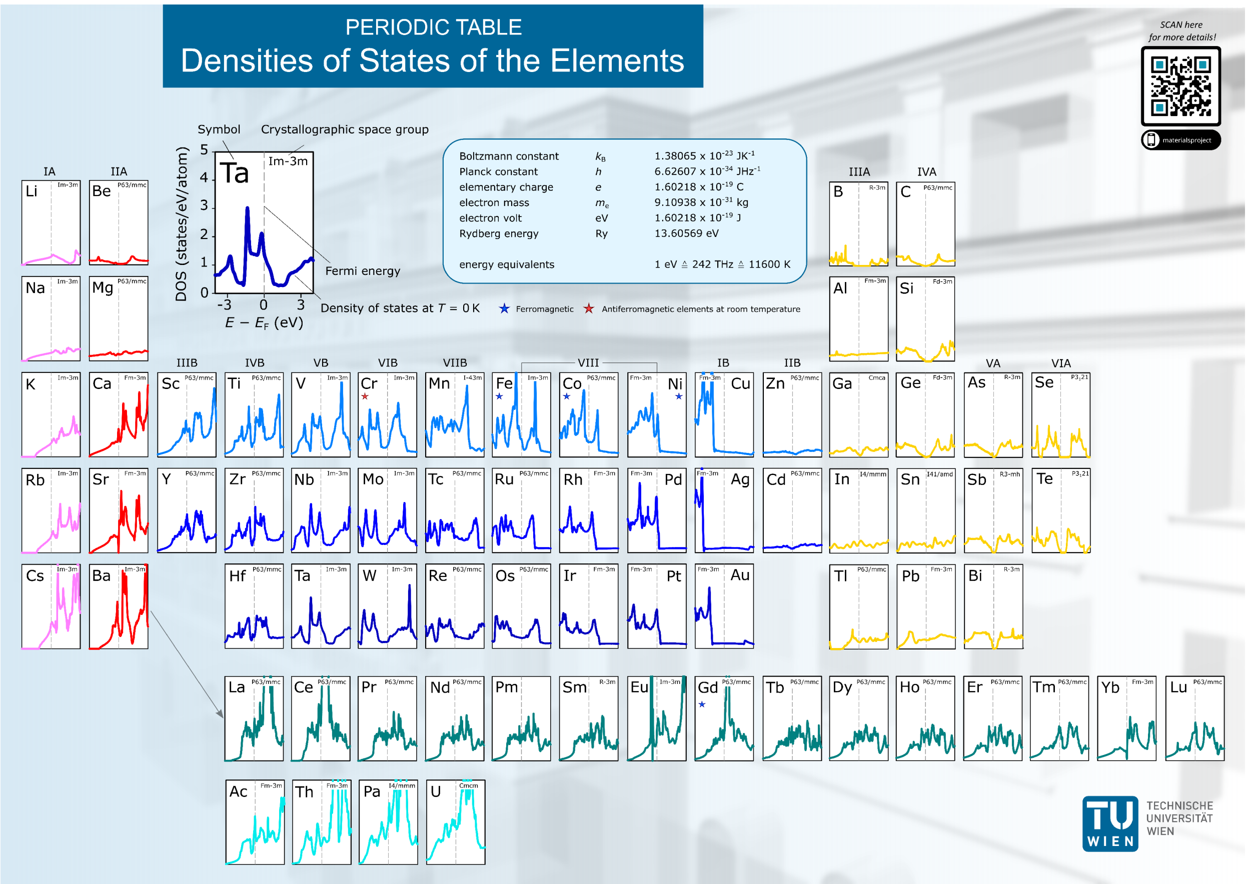

fig. S1. Periodic table of densities of states of the elements. Densities of states as a function of energy near the Fermi level for the different elements of the periodic table. Computational data were taken from the Materials Project database for the experimentally observed room-temperature crystal structure under ambient conditions.

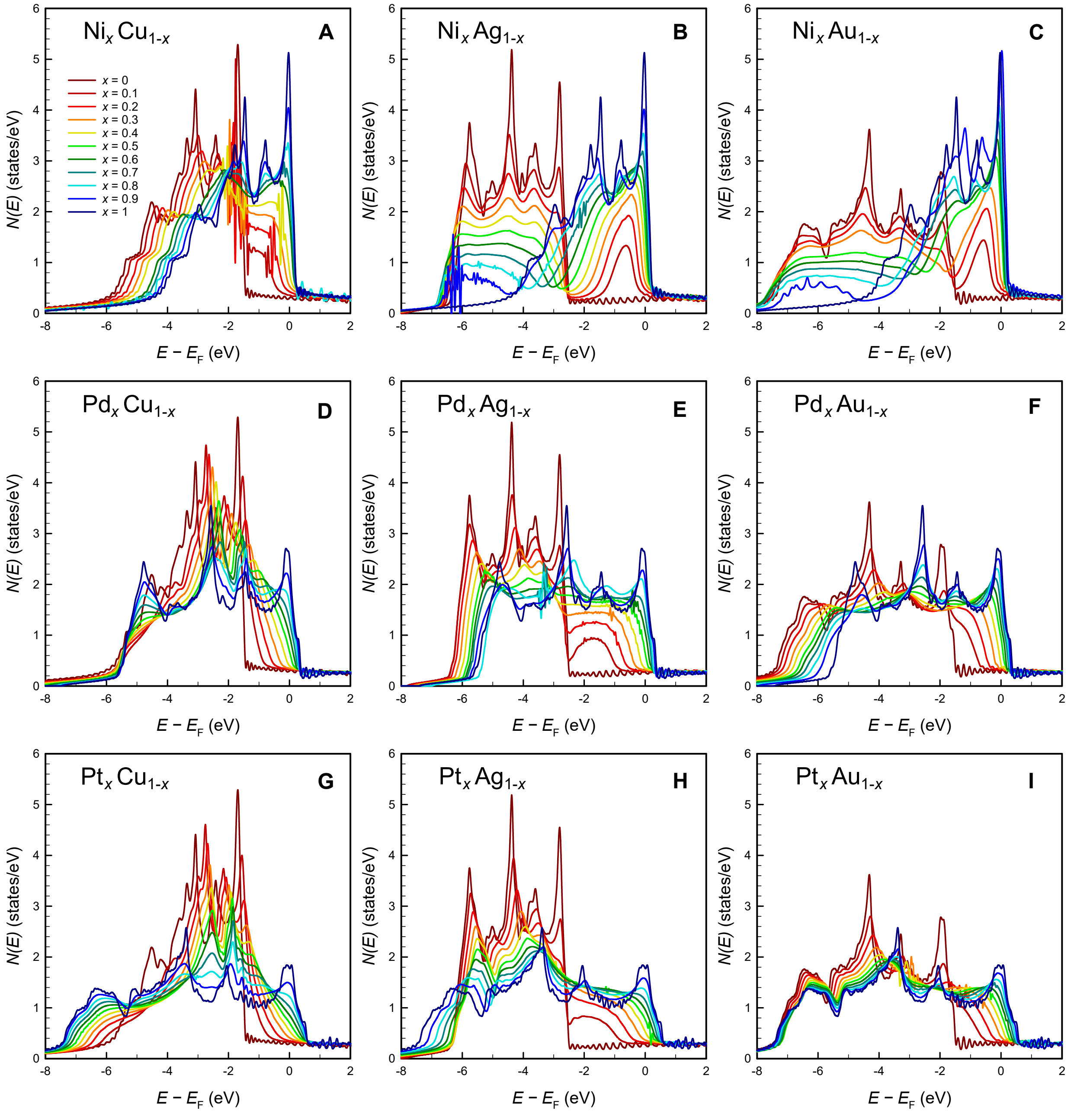

fig. S2. Densities of states of binary metallic alloys comprising group 10 and 11 transition metals. (**A** to **I**) Alloy-averaged densities of states of binary alloys from transition metals of group 10 with transition metals of group 11 elements; calculated for different alloy concentrations.

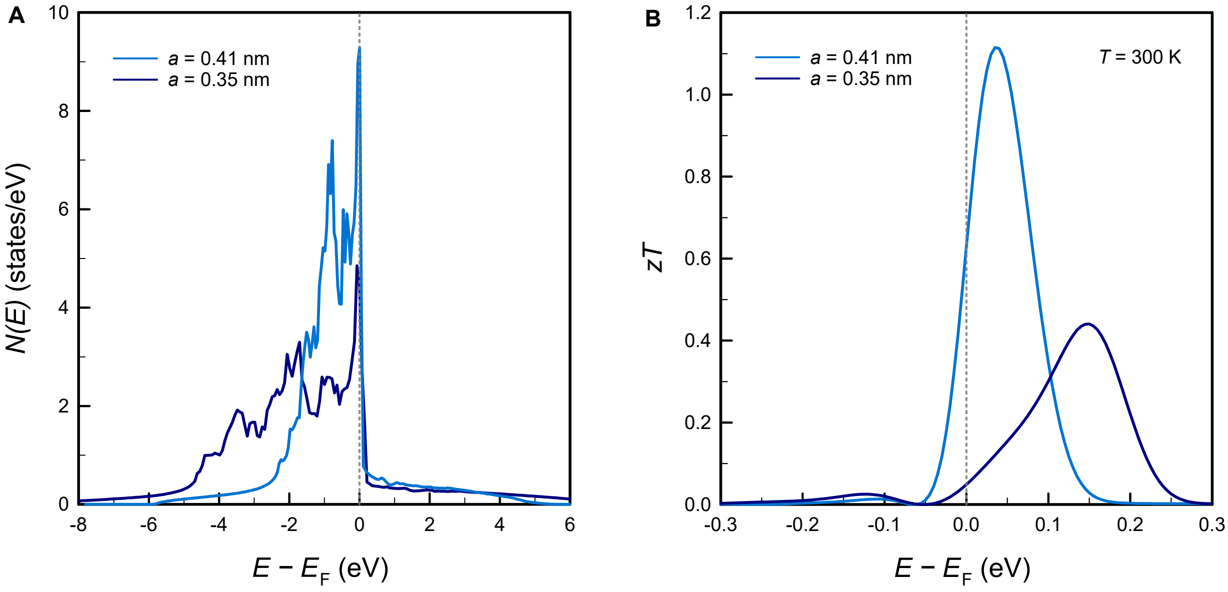

fig. S3. Bandwidth-dependent thermoelectric performance of pure nickel. (**A**) Densities of states of Ni, calculated for different lattice parameters (i) the experimental lattice parameter of Ni and (ii) for a fictitious Ni crystal with a $\approx 16\,\%$ larger lattice parameter (same as the one of pure Au). The localization of the Ni $3d$ states increases as the bandwidth decreases with increasing lattice parameter. (**B**) Dimensionless figure of merit $zT = S^2/L$ corresponding to the densities of states in the left panel; calculated at room temperature by solving the transport integrals for different positions of the Fermi energy.

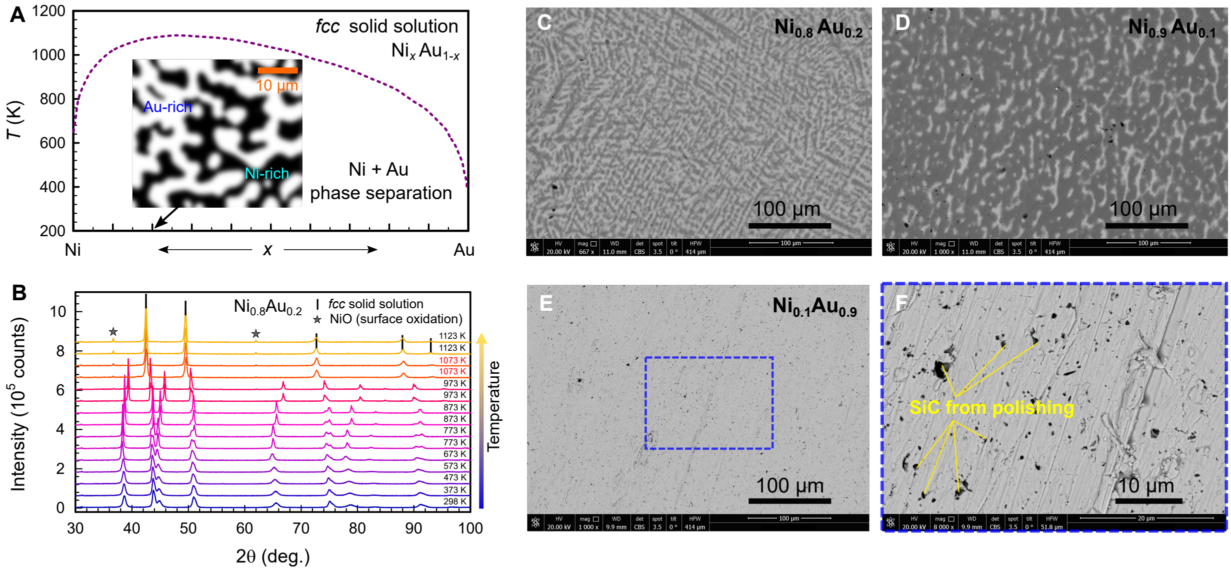

fig. S4. Phase diagram and structural properties of the Ni-Au system. (**A**) Phase diagram of the binary Ni-Au system, showing a large miscibility gap and narrow region of solid solubility at high temperatures. Inset shows a high-contrast scanning electron microscopy image for a slowly cooled two-phase NiAu sample. (**B**) X-ray diffraction patterns at various temperatures for a two-phase $Ni_{0.8}Au_{0.2}$ sample, which transitions into a single-phase fcc alloy at high temperatures. Each diffraction pattern was obtained within a time scale of approximately 30 minutes. (**C** to **D**) Two-phase microstructure of slowly-cooled Ni-rich alloys. (**E**) Single-phase microstructure of a Au-rich $Ni_{0.1}Au_{0.9}$ alloy. (**F**) Holes and SiC inclusions from the cutting and polishing of the sample due to the soft and ductile nature of Au-rich alloys.

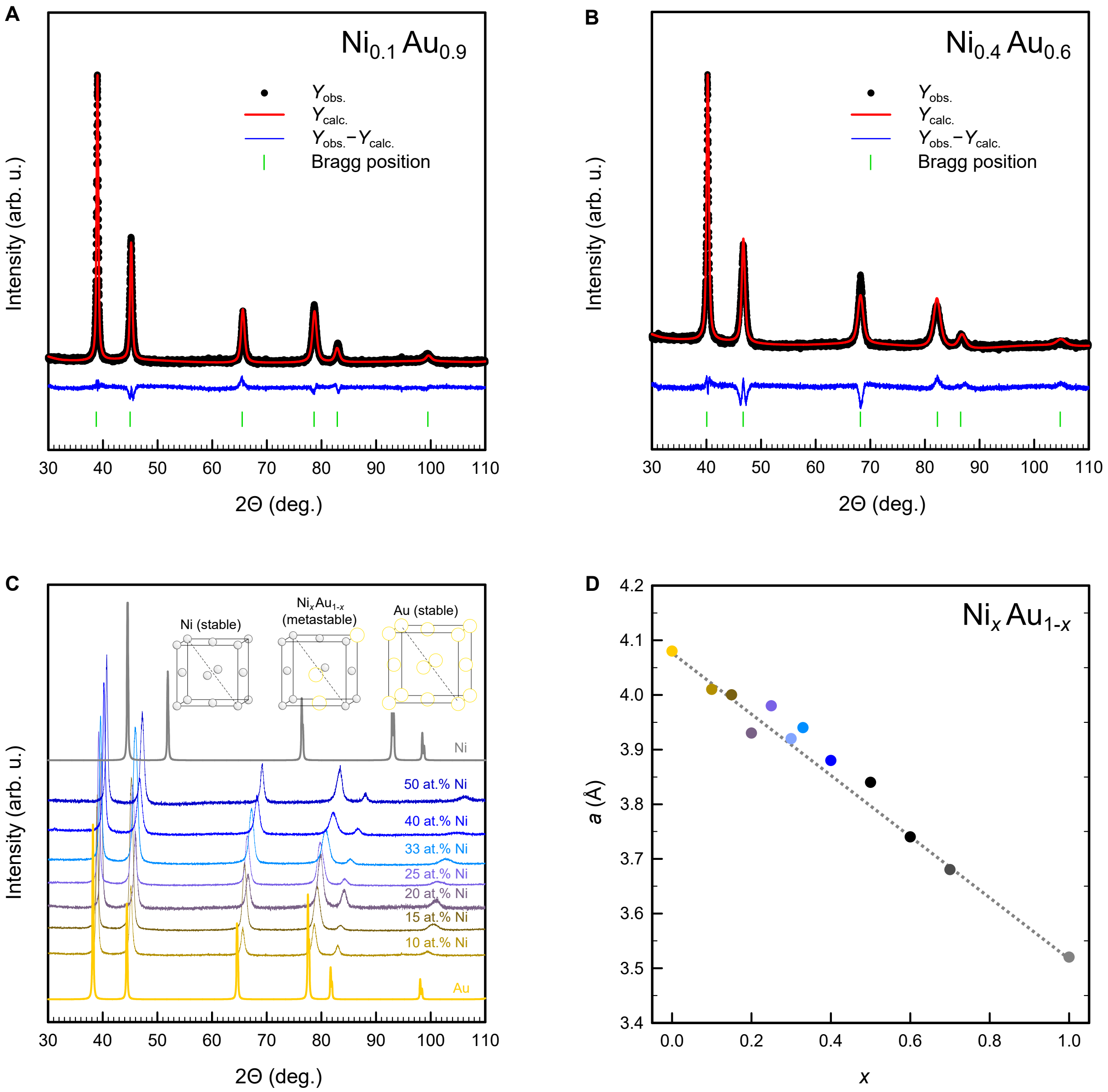

fig. S5. Crystal structure of rapidly cooled NiAu alloys. X-ray diffraction patterns, Rietveld refinement, and difference between model and experimental data for (**A**) quenched $Ni_{0.1}Au_{0.9}$ and (**B**) $Ni_{0.4}Au_{0.6}$. (**C**) X-ray diffraction patterns of numerous NiAu alloys with high thermoelectric performance, which were rapidly cooled from the melt to retain a single fcc phase (**D**) Lattice parameters of the rapidly cooled Ni-Au alloy system as a function of Ni concentration.

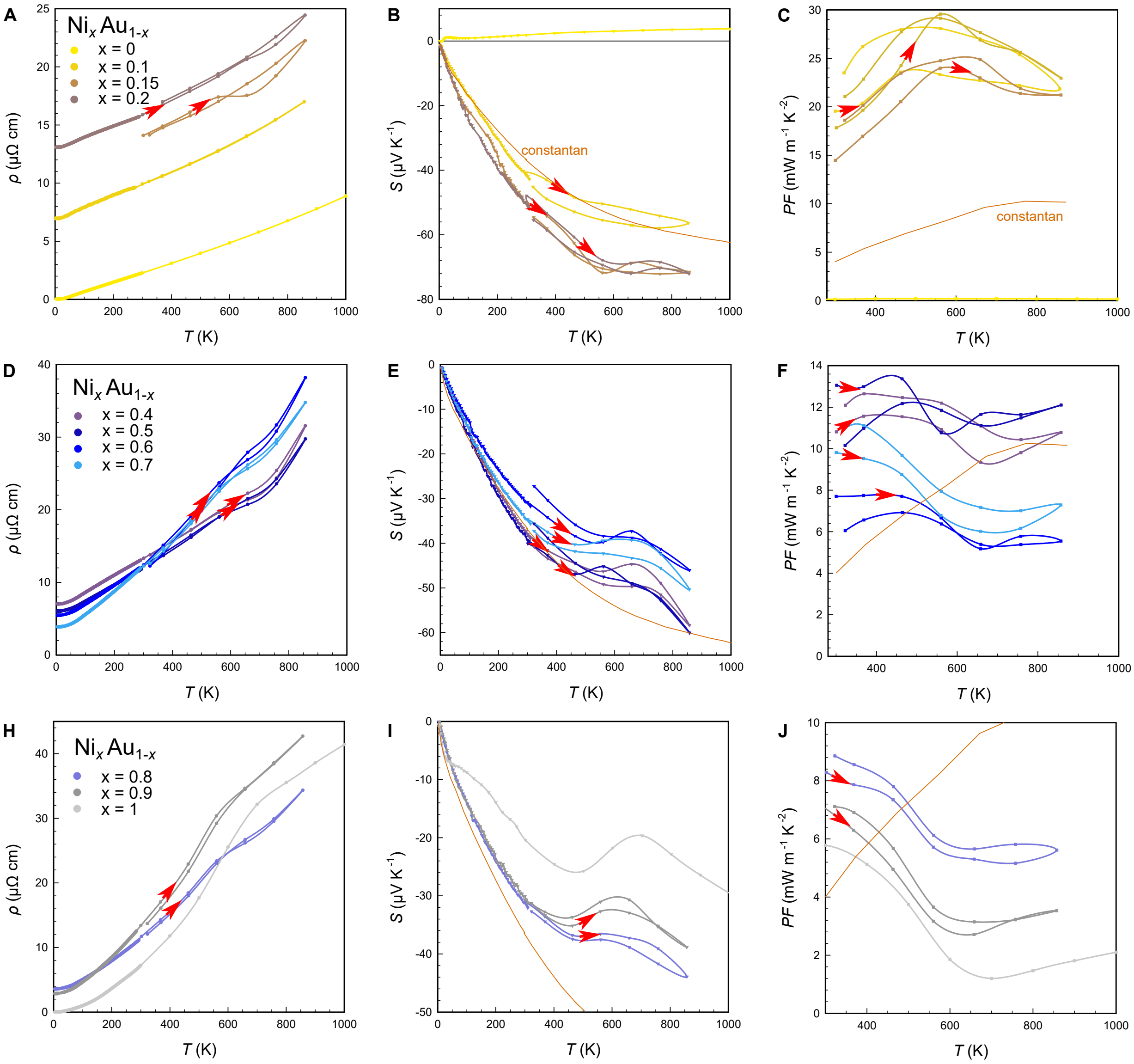

fig. S6. Thermoelectric properties of slowly cooled NiAu alloys. (**A**) Electrical resistivity (**B**) Seebeck coefficient (**C**) power factor as a function of temperature. Red arrows indicate measurement curves upon heating. Slowly cooled Ni_xAu_{1-x} alloys display a two-phase microstructure consisting of a Au-rich phase and a Ni-rich phase of almost pure Ni.

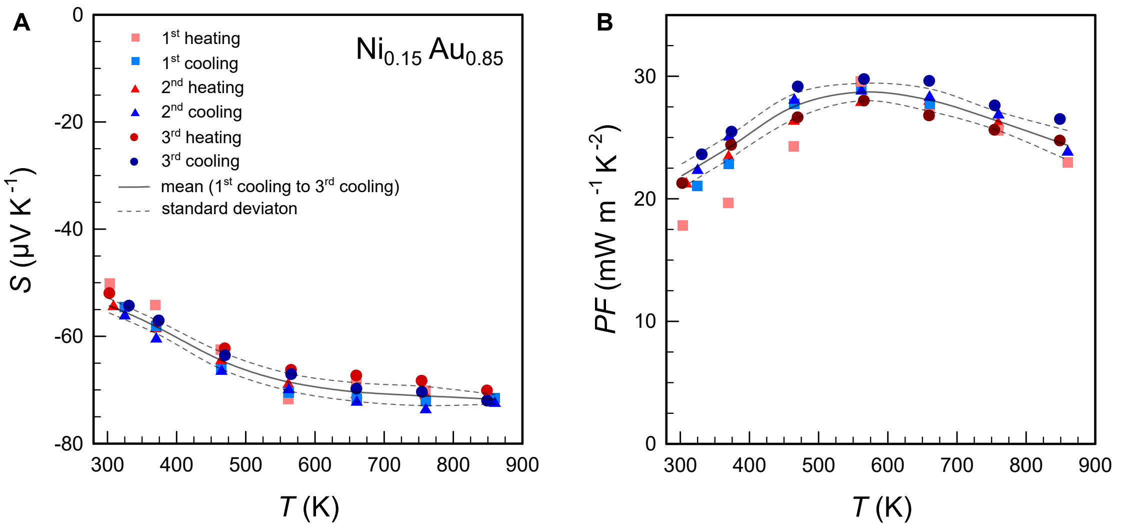

fig. S7. Thermal stability and reproducibility of the thermoelectric properties of $Ni_{0.15}Au_{0.85}$. (**A**) Seebeck coefficient (**B**) power factor of $Ni_{0.15}Au_{0.85}$ as a function of temperature for three consecutive measurements with one heating and one cooling cycle each. During the first heating cycle a subtle change in the thermoelectric properties is apparent, arising from slight changes in the microstructure and composition due to the metastable nature of the alloy. Consecutive measurements yield good agreement and reproducibility with $< 3\,\%$ relative standard deviation from the mean for the Seebeck coefficient and $< 5\,\%$ for the power factor. Solid lines show the mean values, calculated from the five measurement curves (1st cooling to 3rd cooling) and dashed lines represent the standard deviation.

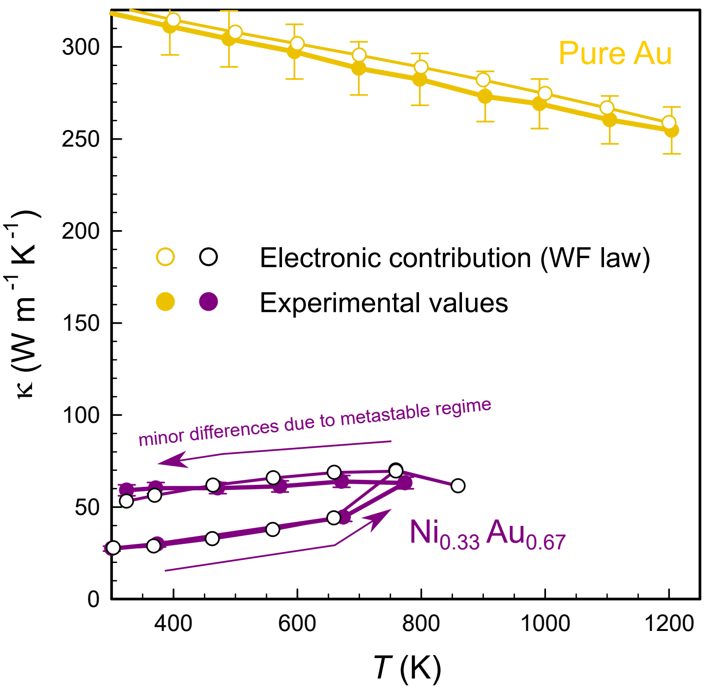

fig. S8. Temperature-dependent thermal conductivity and validity of Wiedemann-Franz law. Experimental thermal conductivity of pure Au and a representative Ni_xAu_{1-x} alloy close to the optimal composition with $x \approx 0.33$ as a function of temperature. It can be seen that both for pure Au as well as for the binary NiAu alloy, the thermal conductivity is entirely dictated by the electron contribution (given by the Wiedemann-Franz law).

Table 1. Thermoelectric data and references for selected metals and state-of-the-art semiconductors shown in Fig. 1B.

Material	Seebeck coefficient $\|S\|$ (μV/K)	$zT_{\max}$	T (K)	References
Aluminum	2.6	0.0003	100	(*29, 44, 53, 54*)
Gold	4	0.0005	1100	(*29, 44, 53, 54*)
Sodium	6	0.0016	300	(*56*)
Copper	7	0.002	1200	(*29, 44, 54*)
Silver	10	0.004	1100	(*29, 44, 54*)
Iron	13	0.007	200	(*34*)
Cobalt	45	0.06	600	(*34*)
Nickel	27	0.026	500	(*29, 34, 44, 54*)
Palladium	37	0.05	1200	(*29, 44, 53–55*)
Platinum	21	0.013	1200	(*29, 57–59*)
Pd_xAu_{1-x}	66	0.2	1200	(*29, 44, 53–55*)
Ni_xCu_{1-x}	73	0.25	1200	(*29, 44, 54*)
Pd_xAg_{1-x}	78	0.28	1200	(*29, 44, 54*)
$YbAl_3$	77	0.19	280	(*32*)
$YbAl_{3-x}Sn_x$	72	0.27	300	(*60*)
Highly doped Si	328–460	0.012–0.026	300	(*61*)
Highly doped Ge	277–346	0.047–0.06	570–770	(*62*)
Si nanowire	240	0.6	300	(*4*)
SiGe	280	1	800	(*63*)
Bi_2Te_3	212	0.7	300	(*64*)
PbTe	270	1.4	750	(*65*)
GeTe	287	2.4	600	(*66*)
SnSe	340	2.6	900	(*5*)
